# Identification and analysis of Single Nucleotide Polymorphisms (SNPs) in the mosquito *Anopheles funestus*, malaria vector

**DOI:** 10.1186/1471-2164-8-5

**Published:** 2007-01-04

**Authors:** Charles S Wondji, Janet Hemingway, Hilary Ranson

**Affiliations:** 1 Liverpool School of Tropical Medicine, Pembroke Place, Liverpool, L3 5QA, UK

## Abstract

**Background:**

Single nucleotide polymorphisms (SNPs) are the most common source of genetic variation in eukaryotic species and have become an important marker for genetic studies. The mosquito *Anopheles funestus *is one of the major malaria vectors in Africa and yet, prior to this study, no SNPs have been described for this species. Here we report a genome-wide set of SNP markers for use in genetic studies on this important human disease vector.

**Results:**

DNA fragments from 50 genes were amplified and sequenced from 21 specimens of *An. funestus*. A third of specimens were field collected in Malawi, a third from a colony of Mozambican origin and a third form a colony of Angolan origin. A total of 494 SNPs including 303 within the coding regions of genes and 5 indels were identified. The physical positions of these SNPs in the genome are known. There were on average 7 SNPs per kilobase similar to that observed in *An. gambiae *and *Drosophila melanogaster*. Transitions outnumbered transversions, at a ratio of 2:1. The increased frequency of transition substitutions in coding regions is likely due to the structure of the genetic code and selective constraints. Synonymous sites within coding regions showed a higher polymorphism rate than non-coding introns or 3' and 5'flanking DNA with most of the substitutions in coding regions being observed at the 3^rd ^codon position. A positive correlation in the level of polymorphism was observed between coding and non-coding regions within a gene. By genotyping a subset of 30 SNPs, we confirmed the validity of the SNPs identified during this study.

**Conclusion:**

This set of SNP markers represents a useful tool for genetic studies in *An. funestus*, and will be useful in identifying candidate genes that affect diverse ranges of phenotypes that impact on vector control, such as resistance insecticide, mosquito behavior and vector competence.

## Background

*Anopheles funestus *and *Anopheles gambiae *are the major malaria vectors in Africa. Due to the difficulty of laboratory colonization, *An. funestus *has not received the same attention as *An. gambiae *and as a consequence there are few molecular markers for this species. However, the recent successful colonization of two strains of *An. funestus *[1] and the identification of a number of microsatellite markers [2,3] have facilitated more detailed studies of this species. Microsatellite markers particularly have been used to study population structure and gene flow between *An. funestus *populations [4-6] and a subset of these microsatellite markers were used to build the first linkage map of this species [7]. However, microsatellite markers are not evenly distributed across the genome, and their low number so far is an obstacle to the development of high resolution linkage maps needed for QTL mapping or association studies in *An. funestus*. Therefore, this study was initiated to increase the availability of characterized and mapped markers for *An. funestus*. Physically mapped ESTs were used to identify SNPs. Such ESTs have been used to study the genetic variability in a number of species such as *Aedes aegypti*, *Drosophila melanogaster *or *Homo sapiens *[8-10] and should be a source of DNA polymorphisms for *An. funestus *as well.

Single nucleotide polymorphisms (SNPs) are by far the most common type of molecular variation in all organisms. They are extremely abundant with an occurrence of about one SNP per kb in human [11] and about one SNP every 125 bp in *An. gambiae *[10]. Significant progress has been made in the development of tools for detection and genotyping of SNPs and they are now becoming the markers of choice for association studies, high-resolution linkage mapping and population genomics studies [12]. SNPs located in non-coding regions of the genome and synonymous SNPs (sSNPs) in coding regions, which have no impact on the phenotype, may provide useful markers for population genetics studies. Non-synonymous SNPs (nsSNPs) which alter the structure (change of amino acid sequence) and potentially the function of encoded proteins are useful markers for association studies to detect genetic variations linked with phenotypic traits.

Patterns of genetic diversity in *An. funestus *have not been studied to the same extent as in *An. gambiae *or *Drosophila *species. Nucleotide diversity in these species has been used to compare patterns of nucleotide variation, such as the relative occurrence of transitions/tranversions in different regions of the genome [8,13]. These surveys have established codon usage and usage bias patterns in many species, with bias hypothesized to occur as a result of selection for efficient translation [14,15].

The sequencing of the 278 million base pairs (Mbp) constituting the *An. gambiae *genome has revealed more than 400,000 SNPs indicating a high level of polymorphism in mosquito species [16]. We hypothesize that by sequencing DNA fragments of different genes of *An. funestus*, a similar level of polymorphism should be encountered and will allow the identification of a significant set of SNPs.

Here, we describe the detection and characterization of a set of genome-wide SNP markers from 50 nuclear genes using two laboratory strains and field samples of *An. funestus*. We also examined patterns of polymorphism and nucleotide diversity in coding and non-coding regions of the genome and define the pattern of codon usage in *An. funestus*. The utility of the SNPs was assessed by genotyping a subset of these SNPs during a linkage mapping study.

## Results and discussion

### Gene amplification

In total, 70 primer pairs were tested by PCR, 55 of which gave reliable amplification with PCR products ranging from 194 to 1342 bp. Sequence data from a total of 21 specimens of *An. funestus *was obtained for 50 of these genes (see Table 1) from laboratory and field samples. Overall, we sequenced a total of 20,547 bp consisting of 14,671 bp of coding region and 5,876 bp of non-coding region. We identified 494 SNPs consisting of 303 coding SNPs (cSNPs) and 191 non-coding SNPs. Each gene contained at least one polymorphism with *BU73 *having one and *BU88 *having 29. The distribution of SNPs among the 50 genes is presented in Table 1. All information concerning the location and the nature of each individual SNP have been submitted to dbSNP, the SNP database of GenBank. These SNPs with their respective reference SNP number (rs) are publicly available in dbSNP Build N°127. The NCBI ss (submitted SNP) numbers of these SNPs are ss65917063 to ss65917416.

### Type of polymorphism

**Figure 1:**
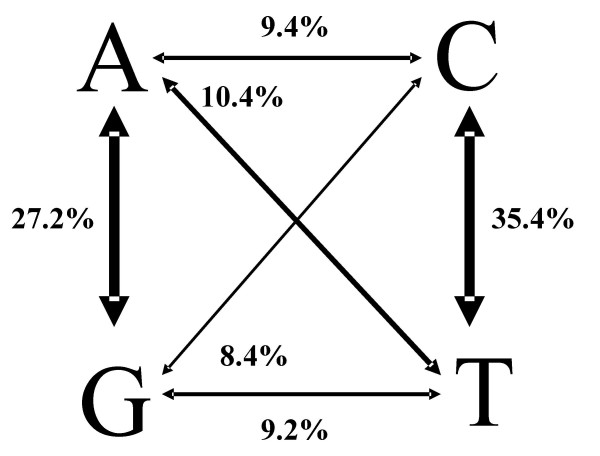
Distribution of transitions and transversions among SNPs.

For all sequenced DNA fragments, transition substitutions were more predominant than transversions (62 % vs 38%). Transitions C↔T and A↔G are over-represented with 35.4 and 27.2 % of the total substitutions respectively while the four transversion classes occurred at similar levels (Figure 1). The higher frequency of C↔T and A↔G SNPs is probably partly related to 5-methylcytosine deamination reactions that occur frequently, particularly at CpG dinucleotides [17]. The preponderance of transitions is more obvious for coding regions where out of the 303 SNPs identified, 201 were transitions (66.3%) and 102 were transversions (33.7%). The ratio of transitions/transversions observed here is close to the 2 : 1 ratio observed for *Drosophila *and humans [13,18]. For polymorphism in non-coding regions, transitions accounted for 55% (106) and transversion for 45% (86). The frequency of transitions between coding and non-coding regions were significantly different (66.3% vs 55% respectively; χ^2 ^= 5.86, *P *< 0.01). This confirms that SNPs occur more frequently as transitions in coding regions than in non-coding regions. There is also a higher frequency of SNPs occurring at the third codon position (63.7%) than at the 1^st ^or 2^nd ^position (Table 1). Similar results have been observed for *Aedes aegypti *[10] and in three species of *Drosophila *[13]. The degeneracy of the genetic code and the selective pressure for gene conservation have been suggested as the main reasons for the preponderance of transitions over transversions [13]. Synonymous or silent substitutions are more often transitions than transversions and there is a stronger selection against replacement substitutions than against synonymous, leading to an increase of the relative frequency of transitions [13]. For fourfold degenerate codons, selection should be neutral, since no amino acid change is induced by a nucleotide substitution at the third position, and each of the 4 codons will produce the same amino acid. We tested this hypothesis by comparing the proportions of transversions at fourfold degenerate codon positions and at non-coding positions for all the 50 genes (Table 2). The result shows that there is no significant difference between the frequency of transversions at fourfold degenerate codon positions (36.8%) and at non-coding regions (44.5%) (χ^2 ^= 2.55 *P *= 0.11), while this difference is significant between coding and non-coding regions (χ^2 ^= 5.33; *P *= 0.021). The fact that fourfold degenerate sites have a similar ratio of transitions/tranversions to non-coding regions is consistent with an hypothesis that the structure of the genetic code and selection against replacement polymorphisms accounts for the preponderance of transition substitutions in coding regions.

Five insertion/deletion polymorphisms (indels) were observed in four genes ranging from 1 to 4 bp in coding, intronic and 5'UTR regions (Table 3). Two indels of 2 and 4 bp were observed in the *BU10 *intron. The frequency of indels (8% for 4/50) is lower than that reported in *Ae aegypti *of 24% [10] or 25% in *An. gambiae *[19]. Only one indel, in the *BU93 *gene, was located in a coding region. This indel was a triplet that did not cause a frame shift. The four indels identified can serve as molecular makers for mapping studies.

Approximately 2/3 (206) of the 303 cSNPs were synonymous substitutions (no modification in amino acid) while around 1/3 (97) were non-synonymous or replacement SNPs leading to a change of amino acid. As approximately two-thirds of random coding substitutions change an amino acid, the fact that only 1/3 of cSNPs are non-synonymous implies strong selection against changes that alter amino acid. This ratio of synonymous and replacement cSNPs is similar to that observed in *An. gambiae *[19] and *Ae. aegypti *[10].

### Genetic diversity

**Figure 2:**
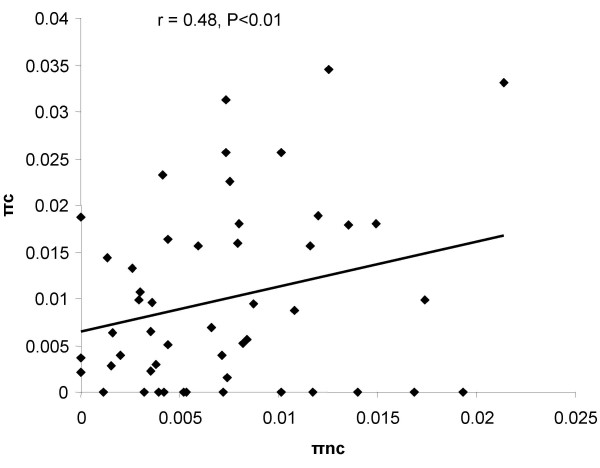
Correlation of nucleotide diversity in coding (π_c_) and non-coding regions (π_nc_) π_c_: nucleotide diversity of coding region, π_nc_: nucleotide diversity of non-coding region.

We estimated the nucleotide diversity for each of the 50 genes in coding and non-coding regions (Table 1). The average nucleotide diversity per gene in coding regions was 7.2 × 10^-3 ^or around 1 SNP every 138 bp similar to that observed in *An. gambiae *(1 SNPs every 125 bp) [19] but much higher than the frequency of 1 SNP/kb observed in humans [20]. SNPs were observed in non-coding regions at a frequency of 1 SNP per 100 bp, corresponding to π = 10 × 10^-3^. Figure 2 shows that there is a positive correlation in the level of polymorphism between coding and non coding regions of *An. funestus *genome within a gene (r = 0.48, *P *< 0.01). This positive correlation may be the consequence of many factors notably the correlated genealogies existing between coding regions and their surrounding non-coding regions. This correlation may also be strengthened by the presence of indirect selection (hitchhiking or background selection) and probably by variable recombination rate, as it is the case in *Drosophila *[21]. Mutational effect of recombination or biased gene conversion can also operate, but this needs to be confirmed as even in *Drosophila*, the effect of biased gene conversion is only suspected but unwarranted [22,23]. The average nucleotide diversity in non-coding DNA (0.010) was lower than in synonymous sites of the coding regions (0.0207), *P *< 0.01. This pattern was also observed in *An gambiae*, *Ae aegypti *and *Drosophila *species [10,13,19]. This is an indication that non-coding regions are under greater purifying selection than synonymous sites within coding regions. This is not surprising, given that non-coding regions may be involved in gene regulation. The non-coding 5'-flanking sequence of a gene may contain regulatory elements such as the promoter that control the expression of that gene, and single-base mutations can affect essential structures for splicing and processing [24].

Nucleotide diversity varies greatly from one gene to another (Table 1) and this is likely related to individual gene function and potentially to differences in selective constraints. However, non-synonymous diversities need to be compared in order to definitely estimate the influence of differences in selective constraints. Among the most polymorphic genes sequenced were cytochrome P450 genes, lysozyme, translation initiation factor and ubiquitin conjugating genes. The non-synonymous nucleotide diversity of these genes varied from 14 to 36.3 × 10^-3^. Most of these genes are involved in specific mechanisms that evolve very rapidly, such as detoxification of xenobiotics for cytochrome P450s or defense mechanisms against bacteria like lysozyme. For example, P450s present a high level of redundancy with less genetic constraints and therefore more polymorphism. In contrast some genes showed very low level of variation particularly those involved in transcriptional or translational regulation (*BU973 *and *BU25, BU93*) or in signaling processes (*BU01, BU08, BU13*). Examples of selective constraints have been observed as well in *Drosophila spp*. where substitution rate between conservative genes and fast evolving genes differ by around 10-fold [25]. Nucleotide diversity was not statistically different between laboratory strains and field collected mosquitoes (7.4 × 10^-3 ^and 6.9 × 10^-3^; *P *= 0.21 by Student's t-test), despite an apparent low level of heterozygosity (fewer heterozygote SNPs) observed in the two laboratory strains compared to the field sample. This result could be due to the fact that FUMOZ and FANG (the two laboratory strains used in this study), were only recently colonized in laboratory and therefore still largely retain the polymorphism of natural populations of *An. funestus*.

The ratio of synonymous to non-synonymous changes (Ka/Ks) gives an indication of the magnitude of the purifying selection against deleterious mutations in a species. The rate of non-synonymous nucleotide substitution per non-synonymous site (Ka) is generally expected to be much lower than the rate of synonymous substitution per synonymous site (Ks), because random amino acid changes are usually deleterious, whereas synonymous changes are likely to be neutral or nearly so [26]. Thus, the expectation is Ka << Ks, except when positive selection is involved favouring particular amino acid replacements, in which case Ka will increase. For *An. funestus *the Ka/Ks ratio was equal to 0.181 and is similar to the ratio of 0.192 observed in *An. gambiae *[19] or 0.204 in *Ae. aegypti *[10] but, higher than the ratio of 0.115 reported in *D. melanogaster *[13]. This result indicates that the purifying selection against deleterious mutations is acting in *An. funestus*. Indeed species with large effective population size such *Drosophila *or *Anopheles *species are generally more effective at purging deleterious mutations [26].

### Clustering pattern of the SNPs

We analyzed the distribution of SNPs identified in this study. We found 16 clusters of two directly neighboring SNPs, one cluster of 3 consecutive SNPs and 13 clusters of two SNPs separated by just 1 bp. For some SNP genotyping methods based on allele-specific amplification, ligation or single base extension principles for which primers need to be designed immediately adjacent to the SNP, it is important that the SNPs are not too close together to prevent primer designing. The presence of a polymorphism within approximately 20 bp will limit the possibilities for designing a robust primer. Most of the 494 SNPs identified in this study do not have a SNP within 20 bp on either or one side thus, and should be easily genotyped by one of these methods.

### Genomic position of the SNPs

Among the 50 genes amplified for SNP detection in this study, 45 are already physically mapped to the *An. funestus *genome by *in situ *hybridization [27], and the remaining 5 genes were genetically located to their respective chromosome by linkage mapping [7]. Overall, 29 SNPs were located on the X chromosome, 334 on chromosome 2 and 131 on chromosome 3. The higher number of SNPs observed on chromosome 2 is also a consequence of the fact that most of the studied genes are located on that chromosome. Table 4 gives the chromosomal location of the 50 genes across the genome of *An. funestus*.

### Polymorphism reliability

To assess the validity of the SNPs identified in this study, 30 SNPs were tested for segregation in isofemale lines. These SNPs were tested using different methods (pyrosequencing, HOLA, SBE and AS-PCR) [7,28]. The Mendelian segregation ratio of each of these SNP loci at F_0_, F_1 _and F_2 _generations was examined in four families from reciprocal crosses between a pyrethroid resistant strain (FUMOZ-R) and a susceptible strain (FANG). Homozygous and heterozygous genotypes for each of these SNPs were observed. Importantly, the expected Mendelian ratio of 1:2:1 was respected in 27 of these 30 SNPs [7], confirming the polymorphism observed at these different positions. We can conclude from this result that the SNPs described in this study are then likely to be true polymorphisms rather than sequence artifacts and our scoring results indicate that they are suitable for use as genetic markers.

### Relevance of the SNPs

The set of SNPs identified in this study provide a very useful tool for future genetic studies in *An. funestus*. These markers are of immediate use for association and QTL mapping studies. Some of these SNPs have been used for linkage mapping and identification of QTL involved in pyrethroid resistance in *An. funestus *[7]. This set of SNPs can be used as tools for population genetic studies in *An. funestus*. Genotyping large number of SNP markers will facilitate the study of genetic structure of natural populations and provide independent estimates of gene flow. It may provide additional markers to study the speciation process observed between the Folonzo and Kiribina chromosomal forms of *An. funestus *[29]. These markers may also be invaluable in monitoring insecticide resistance genes or genes involved in vector competence.

## Conclusion

Through the sequencing of DNA fragments from 50 genes of *An. funestus*, we identified a set of 494 SNP markers and studied the pattern of genetic variability in this species. The distribution of SNPs in *An. funestus *was not neutral but under the influence of regional factors such as recombination, the degeneracy of the genetic code and selective constraints for gene conservation. The SNP markers described constitutes an important resource for more genetic studies in this important malaria vector.

## Methods

### Mosquito samples used for polymorphism discovery

We used adult female specimens of *An. funestus *from two laboratory strains, FANG and FUMOZ-R (seven specimens for each strain) as well as seven field specimens. FANG is a pyrethroid susceptible strain from Calueque, southern Angola and FUMOZ-R is a pyrethroid resistant strain from southern Mozambique [1]. Field specimens of *An. funestus *were collected from Kela village in Chikwawa district in southern Malawi.

### Selection of gene sequences for SNP identification

**Figure 3:**
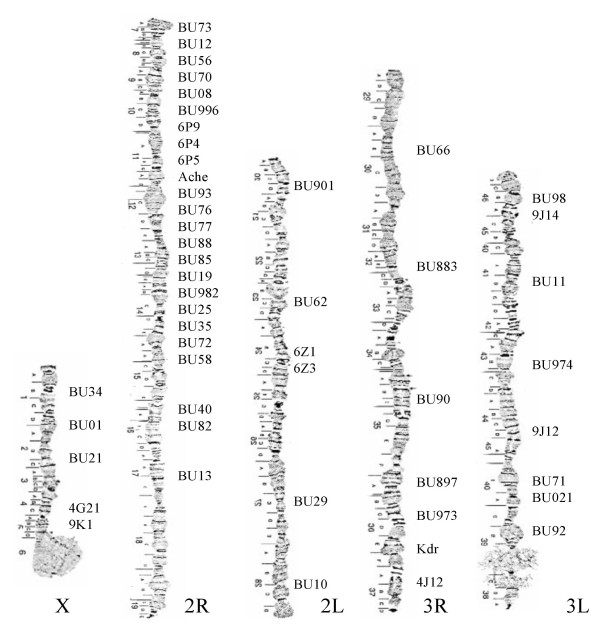
Relative location of studied genes on the *An. funestus *genome. For definitions of genes, see Table 4. This figure was adapted from [37].

Target genes were selected among cytochrome P450 genes for their putative involvement in insecticide resistance [30] or among genes of a broad range of functions that had been physically mapped to *An. funestus *polytene chromosomes [27] (see Table 4; Figure 3). They were also chosen to be distributed across the genome of *An. funestus*. The sequences of the physically mapped cDNAs were retrieved from Genbank. Determination of coding sequence, UTRs and intronic regions were done using the BLAST procedure through NCBI.

### Gene amplification and sequencing

Genomic DNA was extracted using the LIVAK method as described previously [31]. Primers were designed using Primer3 software [32] to flank putative intron sites to maximize the chance of SNP identification. Genomic DNA from 21 individuals (7 from FUMOZ-R, 7 from FANG and 7 from Kela) was amplified for each gene. PCR was performed with 10 ng of genomic DNA in a final volume of 25 μl containing, 2.5 μl Taq buffer, 0.2 mM of dNTPs, 10 pmoles of each primer, 2.5 mM of MgCl2, 0.2 unit of Taq polymerase (Qiagen). Amplification was performed with the following conditions: 1 cycle at 94°C for 3 min; 35 cycles of 94°C for 30 s, 57°C for 30 s and elongation at 72°C for 30 s; followed by 1 cycle at 72°C for 10 min. The annealing temperature was optimized for each primer pair and varied between 53°C to 62°C.

PCR products were purified using the QIaquick PCR purification kit (Qiagen) and directly sequenced on both strands using a Beckman CEQ 8000 automatic sequencer.

### Sequence analysis and SNP detection

SNPs were detected as sequence differences in multiple alignments using Clustalw [33]. Electrophoregrams were visually inspected using BioEdit and heterozygotes were identified [12]. SNPs were identified as transitions or transversions in coding and non-coding regions. SNPs located within coding regions were classified as synonymous or non-synonymous and their codon position determined. Nucleotide diversity analyses were performed using DnaSP 4.0 [34]. The average number of nucleotide substitutions per site between two sequences, π was calculated for each gene as well as the haplotype diversity. The average number of synonymous substitutions per synonymous site (Ks) and non-synonymous substitutions per non-synonymous site (Ka) was computed according to [35].

### SNP validation

Many of the SNPs discovered in this study were validated by different methods. As a part of an effort to construct a genetic map and to identify QTL involved in pyrethroid resistance, 30 SNP loci were genotyped in several families generated from a cross between FANG and FUMOZ-R strains of *An. funestus*. These SNPs were scored using a HOLA (Hot Oligonucleotides Ligation Assay) method [36], single base extension (SBE) using Beckman CEQ8000 and a pyrosequencing method [7].

## Authors' contributions

CSW (corresponding author) carried out the experiments; analyzed the data and wrote the manuscript. JH is the PI of the program that funded the work and contributed to the critical review of the draft manuscript. HR contributed to the design of the study and critical review of the draft manuscript. All authors read and approved the final manuscript.

## Figures and Tables

**Table 1:** Nucleotide polymorphism in *An. funestus *genes

		**Coding region**																**Non-coding region**				
		
		**Polymorphic sites**												**Nucleotide diversity**				**Polymorphic sites**				**Nucleotide diversity**
											
			**Transition**				**Transversion**															
**Gene**	**nHap**	**L (bp)**	**1st**	**2nd**	**3rd**	**Σ**	**1st**	**2nd**	**3rd**	**Σ**	**Syn**	**Rep**	**Σ**	**π**	**π**_n_	**K**_s_	**K**_a_	**L(bp)**	**T**_s_	**T**_v_	**Σ**	**π**

***4G17***	3	190	0	1	1	2	0	0	0	0	1	1	2	0.0052	0.0036	0.0200	0.0072	0	0	0	0	0.0000
***9K1***	23	199	3	1	3	7	1	0	1	2	5	4	9	0.0193	0.0107	0.1010	0.0270	0	0	0	0	0.0000
***4J10***	14	141	1	0	3	4	0	0	1	1	5	0	5	0.0116	0.0000	0.1300	0.0000	73	2	2	4	0.0157
***6P3***	18	0	0	0	0	0	0	0	0	0	0	0	0	0.0000	0.0000	0.0000	0.0000	179	6	2	8	0.0188
***6P4***	17	201	0	0	2	2	0	0	0	0	2	0	2	0.0041	0.0000	0.0450	0.0000	189	8	3	11	0.0233
***6P5***	5	189	0	0	1	1	0	0	0	0	1	0	1	0.0016	0.0000	0.0200	0.0000	112	2	0	2	0.0063
***6Z1***	14	444	1	0	5	6	0	0	5	5	8	3	11	0.0079	0.0022	0.0750	0.0088	29	1	0	1	0.0159
***6Z3***	14	453	1	0	13	14	1	0	5	6	18	2	20	0.0169	0.0011	0.1860	0.0057	0	0	0	0	0.0000
***9J12***	9	156	2	1	0	3	1	0	1	2	0	5	5	0.0117	0.0156	0.0000	0.0510	0	0	0	0	0.0000
***9J14***	14	174	0	0	3	3	2	0	0	2	3	2	5	0.0140	0.0077	0.0760	0.0150	0	0	0	0	0.0000
***BU01***	2	162	0	0	1	1	0	0	0	0	1	0	1	0.0011	0.0000	0.0230	0.0000	26	0	0	0	0.0000
***BU08***	12	394	0	0	2	2	2	1	2	5	4	3	7	0.0042	0.0026	0.0470	0.0098	16	0	0	0	0.0000
***Ache***	18	249	0	1	7	8	0	1	2	3	8	3	11	0.0149	0.0034	0.1460	0.0160	85	2	2	4	0.0180
***BU10***	16	294	1	0	3	4	0	0	2	2	5	1	6	0.0084	0.0024	0.0690	0.0045	245	3	1	4	0.0056
***BU11***	4	177	0	1	0	1	0	0	0	0	0	1	1	0.0029	0.0039	0.0000	0.0074	53	0	1	1	0.0099
***BU12***	7	504	1	1	1	3	0	1	1	2	3	2	5	0.0032	0.0011	0.0240	0.0053	38	0	0	0	0.0000
***BU13***	19	363	2	0	1	3	0	0	0	0	1	2	3	0.0026	0.0029	0.0110	0.0073	148	2	5	7	0.0133
***BU19***	22	526	3	1	4	8	0	1	1	2	6	4	10	0.0072	0.0035	0.0490	0.0100	0	0	0	0	0.0000
***BU021***	17	267	0	0	2	2	0	0	2	2	4	0	4	0.0044	0.0000	0.0610	0.0000	67	2	2	4	0.0164
***BU21***	16	417	0	1	2	3	1	0	2	3	4	2	6	0.0073	0.0018	0.0510	0.0077	81	4	1	5	0.0256
***BU25***	2	234	0	0	0	0	0	0	0	0	0	0	0	0.0000	0.0000	0.0000	0.0000	111	2	0	2	0.0036
***BU29***	11	258	0	0	2	2	0	0	2	2	3	1	4	0.0038	0.0008	0.0540	0.0049	152	0	2	2	0.0029
***BU34***	11	231	0	0	2	2	0	2	2	4	4	2	6	0.0101	0.0051	0.0740	0.0113	28	0	0	0	0.0000
***BU35***	12	264	0	0	7	7	0	0	1	1	8	0	8	0.0071	0.0000	0.1280	0.0000	108	1	1	1	0.0039
***BU40***	14	255	2	1	1	4	1	0	0	1	1	4	5	0.0074	0.0076	0.0170	0.0200	415	3	2	5	0.0016
***BU56***	4	156	0	1	2	3	1	0	2	3	3	3	6	0.0214	0.0132	0.0710	0.0263	126	5	2	7	0.0331
***BU58***	7	261	1	0	2	3	0	1	1	2	3	2	5	0.0053	0.0019	0.0446	0.0103	20	0	0	0	0.0000
***BU62***	22	213	0	0	1	1	0	0	0	0	1	0	1	0.0013	0.0000	0.0207	0.0000	260	7	5	12	0.0143
***BU66***	25	282	9	2	0	11	1	2	0	3	3	11	14	0.0174	0.0194	0.0503	0.0494	179	4	4	8	0.0099
***BU70***	5	228	0	0	2	2	0	0	2	2	2	2	4	0.0066	0.0015	0.0506	0.0059	62	0	1	1	0.0069
***BU71***	9	573	0	0	6	6	1	1	2	4	8	2	10	0.0044	0.0005	0.0615	0.0045	98	1	0	1	0.0051
***BU72***	20	354	2	1	2	5	2	1	1	4	3	6	9	0.0082	0.0062	0.0368	0.0220	195	1	1	2	0.0052
***BU73***	5	277	0	0	0	0	1	2	0	3	0	3	3	0.0039	0.0036	0.0000	0.0135	0	0	0	0	0.0000
***BU76***	15	330	0	0	7	7	1	1	1	3	9	1	10	0.0087	0.0008	0.1104	0.0041	154	3	3	6	0.0095
***BU77***	18	261	3	0	1	4	1	2	0	3	6	1	7	0.0125	0.0136	0.0166	0.0298	198	9	7	16	0.0345
***BU82***	13	228	0	0	2	2	0	2	0	2	2	2	4	0.0059	0.0025	0.0402	0.0112	88	2	2	4	0.0156
***BU85***	14	299	2	1	8	11	0	0	1	1	11	1	12	0.0108	0.0363	0.1155	0.0033	121	2	0	2	0.0087
***BU88***	16	441	2	0	12	14	0	0	2	2	16	0	16	0.0135	0.0000	0.1601	0.0000	212	9	4	13	0.0179
***BU90***	4	0	0	0	0	0	0	0	0	0	0	0	0	0.0000	0.0000	0.0000	0.0000	261	1	2	3	0.0021
***BU92***	11	345	3	1	3	7	1	1	1	3	5	5	10	0.0075	0.0031	0.0650	0.0186	110	5	3	8	0.0226
***BU93 ***	8	510	0	0	5	5	0	0	3	3	7	1	8	0.0035	0.0004	0.0626	0.0025	137	0	2	2	0.0023
***BU98***	4	369	0	0	5	5	0	0	2	2	7	0	7	0.0101	0.0000	0.0843	0.0000	158	2	6	8	0.0257
***BU883***	21	354	1	0	5	6	2	1	2	5	7	4	11	0.0120	0.0052	0.0913	0.0145	188	8	3	11	0.0189
***BU897***	19	303	0	0	2	2	3	1	2	6	3	5	8	0.0080	0.0078	0.0458	0.0210	167	4	3	7	0.0180
***BU901***	12	231	0	0	3	3	0	0	0	0	3	0	3	0.0035	0.0000	0.0552	0.0000	229	2	2	4	0.0065
***BU973***	5	471	1	1	0	2	0	0	0	0	2	0	2	0.0015	0.0019	0.0000	0.0054	67	1	0	1	0.0028
***BU974***	3	459	0	0	2	2	0	0	1	1	3	0	3	0.0036	0.0000	0.0265	0.0000	52	1	0	1	0.0096
***BU982***	4	240	0	1	1	2	0	0	0	0	1	1	2	0.0030	0.0016	0.0190	0.0053	51	1	0	1	0.0107
***BU996***	18	543	4	0	2	6	1	1	1	3	6	3	9	0.0073	0.0019	0.0518	0.0070	87	6	6	12	0.0312
***Kdr***	13	201	0	0	0	0	1	1	0	2	0	2	2	0.0020	0.0026	0.0000	0.0127	501	2	5	7	0.0039

**Total**		**14671**	**45**	**17**	**139**	**201**	**25**	**23**	**54**	**102**	**206**	**97**	**303**					**5876**	**106**	**85**	**191**	

**Average**														**0.0072**	**0.0041**	**0.0537**	**0.0097**					**0.0123**

nHap, number of haplotypes; L, length of the nucleotide sequence; ∑, total; Syn, synonymous substitutions; Rep, replacement substitutions; π, average number of nucleotide substitution per site; π_n_, average number of non-synonymous nucleotide substitution per site; K_s_, average number of nucleotide substitution per synonymous site; K_a_, per non-synonymous site; T_s_, transitions; T_v_, transversions.

**Table 2:** Transition (T_s_) and transversion (Tv) polymorphisms for different classes of DNA

	**Polymorphism**			**Probability**		
	**T_s_**	**T_v_**	**%T_v_**	**Coding Region**	**3^rd ^coding position**	**Fourfold**

**Non coding regions**	106	85	44.5	*P *= 0.021	*P *= 0.0	*P *= 0.11
**Coding regions (Cd-R)**	201	102	33.6		*P *= 0.204	*P *= 0.507
**Third coding position**	139	54	27.9			*P *= 0.047
**Fourfold degenerate sites**	60	35	36.8			

**Table 3:** Indel polymorphism

		**Non coding region**		
**Gene**	**Coding region**	**Introns**	**5' UTR**	**3'UTR**

*6P5*		4 bp		
*BU10*		2 bp, 4 bp		
*BU66*			1 bp	
*BU93*	3 bp			

**Table 4:** Characteristics of genes amplified for SNP detection

**Genes**	**Chromosomal Location**	**Accession no.**	**Function**	**Forward primer**	**Reverse primer**	**Product length**	**No of SNPs**
***4G21***	X	AY648704	Cytochrome P450	GGCGATAGCAAACGTAAAGC	CGCGGTAAACGGAATATAGC	303	**2**
***9K1***	X	AY987362	Cytochrome P450	GTACGAGCTGGCCGTTAATC	CCTTTCTGTAGCTGCACCTTG	243	**9**
***4J12***	3R	AY648706	Cytochrome P450	CCAACAAATCAGTTCATCAGC	TTGTAAAAGTGCTTAAAATG	270	**9**
***6P9***	2R: 9A-12C	AY729661	Cytochrome P450	GCGCCTTAGACAAGAGATCA	AAGGGATGTCGCTTCTTCTC	350	**8**
***6P4***	2R: 9A-12C	AY987359	Cytochrome P450	GTACGAGACTGGCAAAGAAT	AAGGAAGACGTATGGATGG	430	**13**
***6P5***	2R: 9A-12C	AY987360	Cytochrome P450	CTGGCTTTGAAACTTCCTC	AGATACACGTAGGGATGTCG	550	**3**
***6Z1***	2L: 25A-27D		Cytochrome P450	ACGATCCGTTCCGGGTAG	GCTAGCGCAGGATACATTCG	550	**12**
***6Z3***	2L: 25A-27D		Cytochrome P450	GACGATCCGTTCCTGAAGAC	ATCGGTAAGCCCGGATATTT	550	**20**
***9J12***	3L	AY729663	Cytochrome P450	TACCGGTGTGCAGCTTGA	CTTTGGCGCGAAGGTAAA	194	**5**
***9J14***	3L	AY729665	Cytochrome P450	CGGACAACGTATGATCGATTT	TTTGGCTTGCATTAAAAGGTG	214	**5**
***BU01***	X:2B	BU039001	type II transforming growth factor-beta receptor	GTGTGTTTGCTTGGGTGTTG	GGCATCGGTAATCAGGATGT	525	**1**
***BU08***	2R:7C-10B	BU038908	rhodopsin	CATTTGTGGAACCCCATTTC	GGTCATTGGTTTACCCGAGA	500	**7**
***Ache***	2R:9C-12C	DQ534435	Acetylcholinesterase	GGGTACGGGACAACATTCAC	CGTTAACGTACGGGTCGAGT	1050	**15**
***BU10***	2L:28A	BU039010	Cyt-c-p-P1	AAGCACAGTTAAACCTTTCG	ACCTAGCCCAATCTCTGTCT	650	**10**
***BU11***	3L:43B	BU038911	protein transporter	ATCTGCTTGCGCTAGATCGT	ATCGCCAAATTTCATCTTCG		**2**
***BU12***	2R:7B	BU038912	Alpha tubilin	AAGCTCGAGTTCGCCATCTA	CTCCAATCCTTTCCGACGTA	800	**5**
***BU13***	2R:15C	BU038913	signal sequence receptor	ACCCTGAGAAATCGTAACAA	CCGATAGTTGAGAGCAATGT	630	**10**
***BU19***	2R:12B	BU038919	Chitinase	CTGTTGCTGCTGCTACATAC	CCGGTCACGTACAAATAGTC	670	**10**
***BU021***	3L:38C-40B	BU039021	Tubilin beta-3 chain	GAGTTGGTTGATGCCGTGTT	CGTCCGGAAACAAATATCGT	400	**8**
***BU21***	X:3A	BU038921	Phosphoribosylaminoimida-zole carboxylase	TTTCAAGGTGAACGGTGTGA	CCATCAAGATGACGACCAGA	475	**11**
***BU25***	2R 12B	BU038925	ferritin heavy chain-like protein	GCGTAAAGCTGTCGTCCTTC	ATTCCCCCGTCAGGTAGTCT	1200	**2**
***BU29***	2L:27B	BU038929	sensory appendage protein	CACCAAGTACGATGGTGTCG	AGGCACTTGGTTTTGCAGTT	410	**6**
***BU34***	X:1C	BU038934	NADH dehydrogenase	GGCAGGTAGCAGCAGTTTTC	CAGTACCAACCGCAACACAC	400	**6**
***BU35***	2R 12B	BU038935	CG6846 gene product	TTCAGCAAACACGTTTCGTC	ACTTGCCCTTGTCCTTGTTG	400	**9**
***BU40***	2R:14B	BU038940	Glutathion peroxydase	AGGCAAAATCAATTTTTGAA	CGTAACAATTTCTCGACCAT	1150	**10**
***BU56***	2R:7B	BU038956	novel An. gambiae salivary protein	AATCTAGAAGCTGCGCCAGA	AATTCTAGGACGGCGATTCC		**13**
***BU58***	2R:12D	BU038958	translation initiation factor	ACTTCCACGCCCAGTGTATC	CGTGCAGAGTTCGAAAACAA	650	**5**
***BU62***	2L:23A	BU038962	cAMP responsive element binding protein	CAATCGGAGCGTAAGGAAAG	CGTTCTCCCGCAAAAACTAA	475	**13**
***BU66***	3R:30A	BU038966	Lysozyme	TAGCTCATAGTGGCGGTTAT	ACTACAACATGTCGTGCAAA	650	**22**
***BU70***	2R:7C	BU038970	Ubiquitin fusion 80	GTGGACTCCGTACCTGGTCA	CTGTAGAATTACAGGAGGGCGTA		**5**
***BU71***	3L:39A	BU038971	structural protein of peritrophic membrane	GGGAAGTCGGTGTAGGGAAT	ACGTTTGGGTCAGGTAGTCG	750	**11**
***BU72***	2R:12B	BU038972	RHO small monomeric GTPase	GATGAAGCTGCCAAAGATCC	TGCCTCGTCGAAAACTTCTT	900	**11**
***BU73***	2R:7A	BU038873	actin binding	AGTAAGAAACGAACGCAAAG	CGGAAAAGTTGGAATGTAAC	430	**3**
***BU76***	2R:10B	BU038976	translation initiation factor	TGCCTACGAACGACGTAATG	GGCTCGTAGCTGGTCACTTC	500	**16**
***BU77***	2R:10C	BU038877	ubiquitin conjugating enzyme	CAACACACTAGCCAGCAAGG	TTTGGTTCGGCCAACATACT	408	**23**
***BU82***	2R:14D	BU038882	Unknown	AGGGCGGTACAACAAAATCT	GCATCGGAGCGTTTCCTA	400	**8**
***BU85***	2R:12E	BU038885	phosphoglycerate mutase	AAAAAGAATGGCCGGAAAGT	CTCATCGCCCAGAATTTCAT	800	**14**
***BU88***	2R:11B	BU038988	translation initiation factor	GTGGCCTCCCACTTTGTTAG	TACCGGATACGGTTGACGAT	800	**29**
***BU90***	3R:35B	BU038990	gustatory receptor	GGGACATCATCATCATCGAC	TTTCGCTTCTCGCGTTAAAT	300	**3**
***BU92***	3L:39A	BU038892	Microtubule binding	CATGCGACCGAAGAGAAGTT	ATCCTGATTCTGGCTCATGG	550	**18**
***BU93 ***	2R:7C-10C	BU038893	prefoldin subunit 2	CACCGGAAACTCGGCTATTA	TATCGGTTCCATCCGAAAAG	550	**10**
***BU98***	3L:46B	BU038898	CG7630 gene product	TGCGTCACCCGTTACAAATA	ACGTGTACGCTTTCCACCTC	550	**15**
***BU883***	3R:32B	BU038883	peritrophin	TTCGTGACACAGTTATACGC	GCACACTTCAGACTTCCTGT	650	**22**
***BU897***	3R:36C	BU038897	NADH dehydrogenase (ubiquinone)	GGGAATTCCGTGATTTTT	GGCAGAAATATCCATAATCG	700	**15**
***BU901***	2L:20C	BU038901	CG18397 gene product	AAAGACACTCCCGCATTACG	CTCGTGTCTGTTTGGCTTGA	480	**7**
***BU973***	3R:36F	BU038973	polyA-binding protein II	AGTAAGAAACGAACGCAAAG	CGGAAAAGTTGGAATGTAAC	630	**3**
***BU974***	3L:40A	BU038974	serine-type peptidase	ACTGGCGGAGAACGTACAAC	TGCTGCACATTAATCAAAGGTT		**4**
***BU982***	2R:12B	BU038982	ferritin 2 light chain homologue	CTAGTTTCCTGTCGCGTTCC	CATCGTCTCCTCCATTACCG	400	**3**
***BU996***	2R:8D	BU038996	vacuolar hydrogen-transporting ATPase	GTTCGCCTACATGTGCTTCA	ACAAAGGGTGTGCAAAAAGG	800	**21**
***Kdr***	3R: 36A-37E	DQ534436	Sodium channel gene	TGCAAAATAGAGTCATTGGTGAA	ATCATCTTCATCTTTGC	1342	**9**
